# Itch in Atopic Dermatitis – What Is New?

**DOI:** 10.3389/fmed.2021.644760

**Published:** 2021-05-07

**Authors:** Franz J. Legat

**Affiliations:** Department of Dermatology, Medical University of Graz, Graz, Austria

**Keywords:** pruritus, itch, atopic dermatitis, biologics, JAK inhibitors

## Abstract

Atopic dermatitis (AD) is among the most frequent inflammatory skin diseases in humans, affecting up to 20% of children and 10% of adults in higher income countries. Chronic pruritus is a disease-defining symptom of AD, representing the most burdensome symptom for patients. Severe chronic pruritus causes significant sleep disturbances and impaired quality of life, as well as increased anxiety, depression and suicidal behavior. Until recently, skin care, topical corticosteroids, and calcineurin-inhibitors were primarily used to treat mild to moderate AD, while phototherapy and immunosuppressive agents such as corticosteroids, cyclosporine, and methotrexate were used to treat patients with moderate to severe AD. The potential short- and long-term adverse events associated with these treatments or their insufficient therapeutic efficacy limited their use in controlling pruritus and eczema in AD patients over longer periods of time. As our understanding of AD pathophysiology has improved and new systemic and topical treatments have appeared on the market, targeting specific cytokines, receptors, or their intracellular signaling, a new era in atopic dermatitis and pruritus therapy has begun. This review highlights new developments in AD treatment, placing a specific focus on their anti-pruritic effects.

## Introduction

AD is one of the most frequent inflammatory skin diseases in humans, affecting up to 20% of children living in higher-income countries. The first signs of the disease usually develop between the 3rd and 6th months of life, and about 60% of cases occur within the first two years of life; 80% of affected children experience symptoms before the 6th year of life. AD is more common in adults than previously thought, with up to nearly 10% affected. These cases result from persistent or recurrent childhood AD or the new onset of AD later in life. Up to 30% of pediatric and 50% of adult AD patients suffer from moderate to severe forms of the disease (1–3). Overall, AD incidence is increasing worldwide, indicating that an environmental factor is contributing to the development of the disease (4).

No biologic marker has yet been defined for the disease; thus, AD is still diagnosed by examining clinical signs and symptoms and using various diagnostic criteria. The most widely used criteria, described by Hanifin and Rajka, define AD with essential, common and associated symptoms (5). These diagnostic criteria use pruritus, eczematous skin lesions, and the chronic or relapsing course of the disease as essential elements to define AD. Upon clinical inspection, eczematous skin lesions observed in patients with a typical age-related distribution may initially attract our attention, but pruritus is usually the “primary symptom” experienced by the affected patients. The point prevalence of chronic pruritus in AD ranges from 87 to 100%, but, in fact, all patients who actively suffer from the disease also suffer from chronic pruritus (6). In mild and moderate forms of AD, patients experience pruritus as the most burdensome symptom overall. But even in severe cases with widespread skin involvement and extensive oozing and crusting, pruritus is still the patients' major concern and a significant burden of the disease (7, 8). In addition to pruritus, patients frequently report experiencing skin pain. For this reason, this topic requires more attention and study in AD patients (9).

Pruritus strongly and negatively impacts the quality of life of affected patients, who complain most frequently about sleep disturbances due to itch. They report that they have difficulties to fall asleep and wake up repeatedly at night, which reduces the overall sleeping time and quality (8). This lack of physical and psychological regeneration at night can considerably reduce daytime attention levels and negatively affect school and work performance levels. The negative effects on the patient's private life and relationships with family and friends are equally significant. Thus, it is not surprising that AD patients with severe pruritus are at higher risk for psychological disorders such as anxiety, depression, and suicidal behavior (8).

Until recenly, topical corticosteroids (TCS) and calcineurin inhibitors (TCI) were the only topical treatments available to treat mild to moderate AD. To treat moderate to severe AD in patients, the only systemic treatments available were phototherapy or photochemotherapy (PUVA) as well as immunosuppressant drugs, such as cyclosporine, methotrexate, azathioprine, or mycophenolate mofetil (10, 11). Acute, severe exacerbations of AD have been and are still treated with systemic corticosteroids, which are associated with a risk of rebound exacerbations after their cessation.

The recent availability of dupilumab, an IL4Ra-antibody, has signaled the beginning of a new era in AD treatment. Based on the increased knowledge of AD pathophysiology, many new substances for topical or systemic treatments of AD are currently in development and being investigated in clinical trials. This will significantly increase our treatment options against both atopic eczematous lesions and chronic pruritus in the near future (12, 13).

## Pathophysiology of Itch in AD

**Figure 1:**
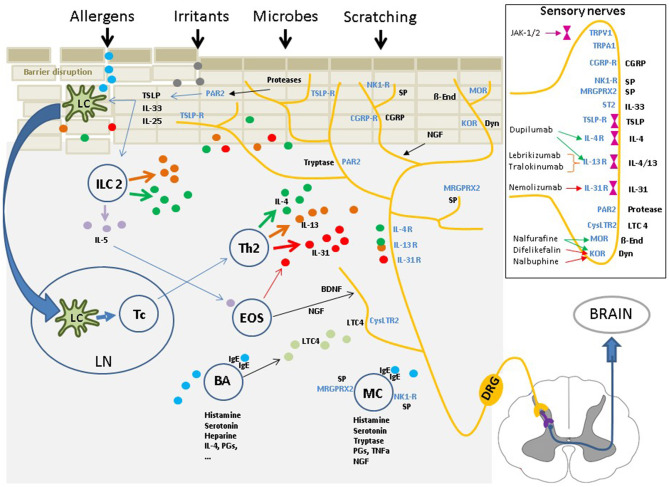
The cutaneous neurosensory system occupies a central position within the “pathophysiological triangle” of epidermal barrier disruption, immune dysfunction, and environmental impacts due to external stimuli. The cutaneous sensory nerves are in close contact with resident and infiltrating cells and are affected by a myriad of mediators from these cells. Upon stimulation, the signal is mediated *via* pruriceptive nerve fibers and the dorsal root ganglia extending to the dorsal horn of the spinal cord. From there, the signal is transferred via interneurons to fibers of the lateral spinothalamic tract, which cross over to the contralateral side, extend up to the thalamus and, finally, reach multiple brain regions, where the nervous signal is perceived as an itching sensation, and scratching is induced. Insert: Multiple itch transmitting receptors are located on sensory nerve fibers, some of which are associated with intracellular Janus kinases. Targeting these receptors or the intracellular Janus kinases with specific inhibitors has shown to have significant antipruritic effects. IL, interleukin; TSLP, Thymic stromal lymphopoetin; NK1-R,neurokinin-1 receptor; CGRP/-R, Calcitonin gene-related peptide /-receptor; MRGPRX2, Mas-related G-protein coupled receptor X2; IgE, Immunoglobulin E; PAR2, Protease activated receptor 2; TRPV1/A1, Transient receptor potential vanilloid 1/ankyrin 1 channel; LTC4, leukotriene C4; CysLTC4, LTC4 receptor; MOR, mu-opioid receptor; KOR, kappa-opioid receptor; Dyn, Dynorphin; ß-End, ß-Endorphin; SP, substance P; ST2, IL-33-receptor.

Genetic predisposition (e.g., filaggrin gene mutation), immune dysfunction and environmental factors (e.g., irritants, allergens, microbiome), and their interactions with each other, play significant roles in AD (1, 2). The cutaneous neurosensory system occupies a central position within this “pathophysiological triangle” of barrier disruptions, immune dysfunction, and external impacts on the skin (Figure 1). Barrier dysfunction within this triangle enables the intrusion of allergens, irritants, or microbial constituents, which eventually stimulate the innate and adaptive immune systems. The immune reactions and released mediators again affect the epidermal barrier, e.g., by reducing filaggrin production (1, 2). Sensory nerves are in close contact with resident and infiltrating skin cells; they can interact intensively with these cells and the mediators released during acute and chronic disease stages (14). Cellular and soluble factors that play a role in eczema development and perpetuation are also important factors in pruritus induction in AD (1, 2, 6). Inflammatory mediators of AD can also sensitize sensory nerves, inducing the phenomena of “hyperknesis” (i.e., increased sensitivity of nerves to pruritic stimuli) and “alloknesis” (i.e., non-pruritic stimuli are perceived as itch). These aspects may contribute to the chronic nature of pruritus in AD (6, 14).

Cutaneous sensory nerves densely innervate all skin layers, including the epidermis, and extend to the stratum corneum. In the skin intercellular spaces, these sensory nerves come in close contact with resident (e.g., keratinocytes, dendritic cells), and infiltrating cells (e.g., lymphocytes, mast cells, eosinophils) and interact with these via a myriad of mediators and receptors (15). These cutaneous sensory nerves in the upper dermal layers include pruriceptive afferent sensory nerves, which convey an itch-signal upon stimulation *via* dorsal root ganglia cells and their central projections to the dorsal horn of the spinal cord. The itch signal is then transferred via interneurons to nerve fibers of the lateral spinothalamic tract, which cross to the contralateral side, and extend to the thalamus. From this point, the signal is distributed to multiple brain regions. In the brain, the signal induces an itching sensation and elicits scratching behavior (16). Researchers have measured an increased density of sensory nerve fibers in skin with AD; therefore, this skin is in a state of neural sensitization, primed to react to signals and interact with the cutaneous environment. An increased concentration of neurotrophins (e.g., the nerve growth factor (NGF) from keratinocytes or the brain-derived neurotrophic factor (BDNF) from neural projections and eosinophils), together with a decreased concentration of the epidermal axon repulsion factor semaphorin A, which is capable of antagonizing the effects of neurotrophins by enhancing nerve sprouting, resulting in hyper-innervation of the inflamed atopic skin (17, 18). This hyper-innervation may eventually lower the threshold for itch induction (i.e., hyperknesis) and favor the induction of itch by non-pruritic stimuli (i.e., alloknesis).

Studies have distinguished histamine-sensitive and histamine-insensitive pruriceptive sensory nerves in the cutaneous neuronal network (14). Antihistaminic drugs have displayed only minor or no effects against pruritus in AD, other than having a soporific effect on patients. This finding indicates that histamine plays only a minor role in AD-associated itch, at least *via* the stimulation of H1 receptors (14). However, histamine may still play a role in AD inflammation and pruritus. Blocking H4 receptors located on immune cells and sensory nerves with specific H4-antagonists had at least some anti-pruritic effects on experimental pruritus (19). Clinical trials, however, showed that no significant reductions in pruritus or eczema occurred in AD patients (20).

These findings show that pruritus in AD is primarily perceived via non-histaminergic sensory nerves. In addition, inflammatory mediators seem to play a central role in AD pathophysiology and can stimulate non-histaminergic sensory nerves, which eventually induces atopic pruritus (14).

### Alarmins and Neuropeptides

These mediators include the so-called alarmins, such as thymic stromal lymphopoetin (TSLP), interleukin (IL)-33, and IL-25. They are released by keratinocytes when they come into contact with various irritants, allergens, or bacterial products (1). Alarmin induction is enhanced when the epidermal barrier is significantly disrupted. In AD, this can be due to an underlying filaggrin gene mutation, the cutaneous inflammation itself, which disturbs the production of epidermal barrier constituents, or by an altered microbiome. In addition, itch-induced scratching also damages the epidermal barrier by mechanically irritating the skin (1, 21).

Irritants, allergens and bacterial products that contact the skin often contain an array of proteolytic enzymes; these can activate protease-activated receptors (PAR) by the proteolytic cleavage of their extracellular N-termini (22). PAR-2 receptors are located on keratinocytes and sensory nerves, and researchers have argued that the stimulation of PAR-2 is a major pathway for non-histaminergic pruritus in AD and the induction of neurogenic inflammation, resulting in the release of neuropeptides such as substance P (SP) and calcitonin gene-related peptide (CGRP) (23). In AD patients, the skin is exposed to various proteolytic enzymes from exogenous (e.g., bacteria or house dust mites) or endogenous sources (e.g., tryptase, trypsin and kallikreins, mainly KLK5) that are released by epithelial or immune cells during inflammation processes, highlighting the importance of this pathway in the early disease phases (24, 25). Recent findings by Zhao et al. (26) revealed intriguingly that PAR-2 activation appears to be located upstream of TSLP and that stimulation of TRPV3 can induce TSLP release. TSLP activates other immune cells, but can also directly stimulate pruriceptive sensory nerve fibers to induce itch (27), a finding that has also been shown for the alarmin IL-33 (28). Thus, keratinocytes could boost and transform irritating stimuli from external or internal sources into itch signals via PAR-2 stimulation and the release of mediators such as TSLP. PAR-2 and TSLP could also be significant “neuro-epidermal communication” elements in AD; in a newly developed mouse model with epidermal overexpression, PAR-2 was recently shown to induce and exacerbate AD signs and symptoms, and especially pruritus (25).

PAR-2, *via* the stimulation of sensory nerves, also induces neurogenic inflammation and the release of neuropeptides such as SP and CGRP (29). SP affects sensory nerves and keratinocytes as well as inflammatory cells (e.g., lymphocytes, mast cells, eosinophils, and basophils) *via* high-affinity neurokinin (NK)-1 receptors and Mas-related G-protein coupled receptors (MrgprX2) (30). Stimulation of MrgprX2 is also involved in SP-induced mast cell degranulation, which stimulates mast cells to release of more inflammatory and pruritogenic mediators, such as histamine, leukotrienes, prostaglandins, TNF-a, proteases, and NGF (30). Interestingly, in a mouse model of acute contact dermatitis, Meixong et al. showed that Mrgprb2 (the murine form of MrgprX2) activation of mast cells differed from classical IgE-mediated stimulation in that it induced a preferential release of tryptase and the eventual induction of non-histaminergic itch, probably *via* PAR-2 activation. Whether MrgprX2 receptors are also mast cell-associated targets in pruritus of AD patients remains to be determined (31).

However, SP can also stimulate pruritus due to the effect of MRGPR-X2 on sensory nerves. This may represent an additional or even the preferred pathway by which SP stimulates pruritus in AD. In part, this may explain why a recent clinical trial in AD patients with the specific NK1R-antagonist serlopitant showed numerical but not significant reduction in pruritus (32), while tradipitant, another NK1-receptor antagonist, slightly but significantly reduced itch in these patients (33). In patients with chronic prurigo, serlopitant also significantly reduced pruritus in a phase-2 study; however, it numerically but not significantly reduced pruritus in two phase-3 studies (32). This indicates that both NK-1 and MrgprX2 receptors obviously play a role in SP-induced pruritus, but the extent to which these two receptors are involved in atopic pruritus in various disease stages requires further evaluation.

The neuropeptide CGRP also affects sensory nerves, blood vessels and immune cells (e.g., dendritic cells and T-cells), facilitates the infiltration of inflammatory cells and propagates a Th2 immune response (15). Similarly, CGRP stimulates the release of IL-13 from circulating CLA+ T-cells in AD; an increased IL-13 release from T-cells has been observed in more severely affected AD patients with more intense pruritus (34). Thus, the neurosensory system – with its antidromic axon reflex of neurogenic inflammation and local release of neuropeptides – occupies the frontline of defense against environmental impacts on the skin, even before the innate or adaptive immune systems are activated.

### Interkeukins (IL)-4, IL-13, and IL-31

The release of the alarmins TSLP, IL-33, and IL-25, together with neuropeptides, not only trigger the induction and perception of itch; they also eventually stimulate the innate and adaptive immune systems. This stimulation initiates and further propagates the predominant Th2 immune response in AD. Subsequently, several pro-inflammatory mediators are released, either directly by type 2 innate lymphoid cells (ILC2) or Th2 effector lymphocytes or indirectly *via* the stimulation of mast cells, basophils, or eosinophils. Many of these mediators can either directly or indirectly stimulate pruritus in AD (1, 2).

The cytokines IL-4 and IL-13 play a central role in AD pathophysiology and also play a significant role in AD itch. These cytokines are produced and released mainly from ILC2 and Th2 cells. By activating specific receptors which share the IL-4Ra chain, they have multiple effects on epidermal and dermal cells as well as on sensory nerve fibers (1, 2). *In vitro* and *in vivo* experiments in mice have underlined the potential of IL-4 and IL-13 to sensitize sensory nerves to itch by lowering the sensitivity thresholds to other pruritogenic stimuli, such as histamine, IL-31 and TSLP (35). However, other studies have shown that both IL-4 and IL-13 also can directly stimulate pruritus in mice and that the application of combinations of these mediators even accelerated itch induction (36).

Involved sensory nerve fibers carry the transient receptor potential (TRP) V1 and TRPA1, which are unspecific cation channels (37). Once these nerves have been stimulated by IL-4, IL-13, or IL-31*via* their specific receptors, the activation of TRPV1 and/or TRPA1 induces calcium influx, which eventually induces the release of action potentials *via* the sodium channels NaV1.7, NaV1.8, or NaV1.9. TRPV1 and TRPA1 must be present for these pruritogens to induce itch or sensitize sensory nerves to other pruritogens (37, 38).

IL-4 and IL-13 also affect the skin barrier and disturb its function by downregulating essential skin barrier proteins such as filaggrin, involucrin, and loricrin (1, 2, 39). This downregulation causes the release of proteolytic enzymes, stimulating PAR-2, and the release of alarmins (IL-25, IL-33, TSLP). This series of events closes a feed-forward loop and fuels atopic inflammation as well as pruritus. IL-4 and IL-13 have also recently been shown to induce the selective expression of kallikrein (KLK)-7 in normal human epidermal keratinocytes. One recent study also implicated KLK-7 in itch induction, regardless of inflammation in AD, *via* an unknown epidermal-neural mechanism (40, 41). These cytokines, however, also affect key immunologic cells such as other lymphocytes, mast cells, eosinophils, and basophils that carry the IL-4 and/or IL-13 receptors. By triggering the release of preformed and newly produced mediators from these cells (e.g., histamine, tryptase, endothelin-1, eotaxin, IL-31), IL-4 and IL-13 further contribute to the stimulation of sensory nerves and the induction of itch in AD (42).

IL-31, the so-called “itch-cytokine” plays a major role in this respect, as it is primarily released by Th2 cells and can directly stimulate specific IL-31 receptors (IL-31R) on sensory nerves, inducing an itching sensation (35). In addition, stimulation of IL-31R, which consists of the IL-31 receptor alpha chain (IL-31Ra) and the Oncostatin M receptor-beta chain, also stimulates the sprouting and branching of these sensory nerves, increasing their sensitivity to IL-31, and other pruritogens (43). By both directly inducing itch and sensitizing nerves to further pruritic stimuli, IL-31 plays a significant role in AD pruritus. This process of neural sensitization by IL-31 may significantly contribute to the development of chronic itch. Specifically, it may play a critical role in the so-called “itch-scratch-cycle,” a phenomenon which strongly promotes the development of highly pruritic nodular chronic prurigo, i.e., prurigo nodularis. Interestingly, high levels of IL-31 have been found in these pruriginous skin lesions (44). Chronic prurigo is frequently associated with atopic diathesis or a history of previous AD, and it can sometimes be found in combination with atopic eczema in AD patients with severe chronic pruritus (45).

### Janus kinase/Signal Transducer and Activator of Transcription

Several of the aforementioned mediators either directly stimulate pruritus or sensitize sensory nerves to other pruritogenic stimuli. Once these mediators have bound to their specific receptors, they transmit their signals via the Janus kinase (JAK)/Signal transducer and activator of transcription (STAT) pathway.

The JAK family has four members: JAK1, JAK2, JAK3, and TYK2 (46). Cytokines that are important for pruritus in AD (e.g., IL-31, IL-4, IL-13, and TSLP) transmit their signals *via* JAK-1 and JAK-2 into the cells. The deletion or inhibition of JAK-1/2 in animal models significantly reduced itch signaling induced by these mediators (35). In humans, JAKs were shown to play a critical role in pruritus when tofacitinib, an oral JAK 1/3 inhibitor, significantly reduced pruritus in elderly patients who suffered from chronic pruritus of unknown origin (35). IL-31, IL-4, IL-13, TSLP, and IL-5, as well as other cytokines influence inflammation and pruritus in AD. The potential to inhibit JAK-1 and JAK-2 with selective JAK-inhibitors opens a new treatment avenue, indicating that it may be possible to block several important itch mediators simultaneously. This avenue should enable us to treat chronic pruritus in AD and other chronically pruritic diseases more effectively (47).

### Opioid System

The central and peripheral opioid system is involved in chronic pruritus. The μ-opioid (ß-endorphin/μ-opioid receptor) and k-opioid (dynorphin A/k-opioid receptor) systems are involved in pruritus modulation (48). Stimulating μ-opioid receptors (MOR) (e.g., with morphine for pain treatment) induces itch, while inhibiting MOR with naloxone or naltrexone or stimulating k-opioid receptors (KOR) with specific agonists can reduce itch (49). Researchers have identified a relative imbalance between the KOR and MOR system with a downregulation of KOR in the epidermis of AD patients. Photochemotherapy (PUVA) could be used to rebalance the deviant KOR/MOR systems by downregulating MOR (its ligand ß-endorphin remained unchanged) and upregulating the reduced dynorphin levels to normal (KOR remained unchanged), leading to itch reduction in AD patients (48). One study found a significant decrease in KOR in patients with end-stage renal disease (ESDR) under hemodialysis, who suffered from chronic itch, as compared to patients without chronic itch, while the expression of KOR correlated significantly and negatively with itch intensity (50). The KOR agonist dynorphin can be used to modulate itch perception by, for example, interacting with KOR on interneurons in the spinal cord (51). Treating ESRD patients who suffered from chronic itch with the KOR agonist nalfurafine had significant antipruritic effects (52). The topical application of nalfurafine also had an antipruritic effect in a murine model of AD (53). Phototherapy can be used to reduce itch in both EDSR and AD patients and may also have an antipruritic effect, at least in part, because it affects the peripheral opioid system in the skin; e.g., the resulting dynorphin release can eventually act on peripheral sites as well as central KOR (54).

## Systemic Treatments

In recent years, scientists have gained a significant amount of knowledge about the pathophysiology and key mediators of inflammation and itch in AD (2). While several agents have been newly developed to treat AD, the results seen *in vitro* and in animal studies still need to be translated from laboratory to the bedside. In the following sub-sections, descriptions of the new treatments are provided. Future studies will provide even further information about whether these agents really be used to improve eczema and chronic pruritus under real-world conditions in daily clinical practice.

### IL-4/IL-13 Blockade

When dupilumab, the first biological agent to be developed to specifically target the IL-4 receptor alpha (IL-4Ra) chain, was licensed in 2017, we entered a new era in AD treatment. Dupilumab inhibits the interactions between IL-4 and IL-13 and their receptors, which share the same IL-4Ra subunit (55).

Two monotherapy studies (SOLO 1 and SOLO 2) clearly showed that dupilumab improved atopic eczematous skin lesions. The agent significantly reached the primary endpoint, reducing the investigator global assessment (IGA) to clear or almost clear and significantly reducing the Eczema Area and Severity Index (EASI) as compared to a placebo. Dupilumab also significantly reduced the weekly average of daily peak pruritus on the numerical rating scale (NRS) from 0 (= no itch) to 10 (= worst imaginable itch) by about 50% as compared to about 20% in controls (56). The significant anti-pruritic effect of dupilumab as compared to the controls was confirmed in subsequent studies (55), and the results of a *post-hoc* analysis of data from four randomized, controlled trials showed that dupilumab could significantly reduce itch by as early as the 2nd day of treatment in adults and the 5th day of treatment in adolescents. Thus, the agent displayed not only good but also rapid itch reduction in AD (57). Similar effects on eczema and pruritus were recently shown with dupilumab treatment in children aged 6–11 years (55, 58).

In addition, dupilumab could significantly reduce pruritus in difficult to treat, highly pruritic diseases such as chronic prurigo and bullous pemphigoid. This finding indicates that IL-4 and IL-13 play important roles in chronic pruritus and that blocking these cytokines could help to relieve pruritus in diseases other than AD (59). However, with dupilumab, it has not yet been possible to determine the relative contributions of IL-4 or IL-13 to these effects. In fact, other researchers have assumed that IL-13 is the primary mediator of AD in peripheral tissues, and some have speculated that dupilumab blocks IL-13 as a primary mechanism of effect in AD (60).

Tralokinumab and lebrikizumab, two biologics that specifically target IL-13, were recently developed, enabling researchers to evaluate the importance of IL-13 in eczema and pruritus in AD. Although, no direct comparisons with dupilumab have been made, both tralokinumab and lebrikizumab significantly reduce eczema and pruritus in AD. In three phase 3 trials, tralokinumab, as monotherapy or in combination with TCS, significantly improved eczema. Tralokinumab also reduced pruritus by ≥ 4 points on the NRS in a significantly higher proportion of patients than in controls, regardless of their concomitant treatment with TCS (45.4 vs. 34.1% in ECZTRA3) or without TCS (20.0 vs. 10.3% in ECZTRA1 and 25.0 vs. 9.5% in ECZTRA2) (61, 62).

Likewise, lebrikizumab also significantly reduced eczema scores in a phase 2b trial. Lebrikizumab was given subcutaneously in a dose of 125 or 250 mg every 4 weeks (with a double loading dose) or in a dose of 250 mg every 2 weeks (without a double loading dose). This treatment enabled significantly more patients to reach a clinically relevant peak-pruritus reduction of ≥4 points NRS as compared to controls (i.e., 41.8, 47.4, and 70.0% as compared to the placebo with 27.3%). Notably, a significant difference in itch reduction was seen as early as day 2 in the high-dose group (63).

Since the study designs differed, we cannot directly compare the effects of tralokinumab and lebrikizumab on pruritus with each other or with the effects of dupilumab. However, these studies clearly show that blocking IL-13 can significantly reduce pruritus in AD. Whether the effects of IL-13 blockade can be enhanced by also blocking IL-4 remains to be determined in a future head-to-head trial with dupilumab, although, it is unlikely that these direct comparisons will be performed very soon.

### IL-31 Blockade

The IL-31Ra antagonist nemolizumab had a highly significant antipruritic effect in patients with moderate to severe AD (64). This study was remarkable, because “peak pruritus” on a numerical rating scale (PP-NRS) was chosen as the primary outcome parameter. Therefore, this challenged the idea that IL-31 was the “primary itch mediator” in AD. In this study, nemolizumab was subcutaneously applied in doses of 0.1, 0.5, or 2.0 mg/kg at baseline and every 4 weeks; significant reductions of 43.7, 59.8, and 63.1%, respectively, as compared to 20.9% by placebo, were seen in peak pruritus over the 12-week trial period (64). Two other placebo-controlled phase 2 trials using fixed regimens confirmed the excellent anti-pruritic effect of nemolizumab (65, 66). In an open-label, long-term extension study of the previous 12-week study, patients were further treated with 0.5 mg/kg of nemolizumab every 4 weeks; the pruritus could be reduced by 89.6% by week 64 (67). The improvement in eczema progressed more slowly than the itch reduction. Thus, EASI was reduced by 47.8% after 12 weeks, but could also be improved by 75.8% after 64 weeks (no control group for comparison) in this long-term extension study (67).

The significant antipruritic effect of nemolizumab could also recently be demonstrated in patients with nodular chronic prurigo (i.e., prurigo nodularis (PN)) (68). PN is a treatment-resistant, distinct disease characterized by severe chronic pruritus, chronic scratching, and pruriginous nodular skin lesions (45). Four weeks after receiving one subcutaneous injection of nemolizumab (0.5 mg/kg), pruritus was reduced by 4.5 points NRS from baseline (i.e., 53.0%) as compared to only 1.7 points (i.e., 20.2%) in placebo-treated prurigo patients. At 12 weeks (i.e., 4 weeks after receiving the last of 3 monthly subcutaneous injections), the itch was even reduced by 61.9% as compared to 25.7% in controls. In addition, the extent of healed nodular skin lesions was significantly better than that seen in controls (68). Phase 3 and long-term extension studies in AD and PN with nemolizumab are currently ongoing; the outcomes of these studies should improve our knowledge about treatment possibilities for these diseases (for AD, ClinicalTrials.gov Identifier: NCT03985943 and NCT03989349 and NCT03989206; and for PN, NCT04501666, NCT04501679, NCT04204616).

These data indicate that, firstly, pruritus in AD and chronic prurigo can be significantly reduced by specifically targeting IL-31, breaking the “itch-scratch-cycle” and eventually enabling nodular prurigo to heal and eczema to improve in AD (64, 68). Secondly, blocking IL-31 with nemolizumab appears more effectively reduce atopic pruritus than eczema (65), while blocking IL-4/IL-13 appears to have a stronger inhibitory effect on eczema than on pruritus (56). To clearly understand the true relative effects of different drugs in specific diseases, these must be compared in head-to-head studies. However, pruritus and eczema in AD have traditionally been believed to be tightly connected with each other, and AD was often described as an “itch that rashes” (69). As new treatments and agents that block specific mediators appear, the regulation of pruritus and eczema in AD may turn out to be more differentiated than previously thought. This knowledge may help us to further customize AD treatments to meet the primary needs of our patients in the future.

### JAK Inhibitors

The findings of Oetjen et al. (35) highlighted the importance of the mediators IL-4, IL-13, and IL-31 for the induction and maintenance of itch in AD. In their experiments, the inhibition of the JAK/STAT pathway, which mediates the intracellular signaling of these cytokines, significantly reduced atopic itch in mice. JAK-1 inhibition displayed especially significant effects on pruritus. In their study, Oetjen et al. (35) also showed that the oral JAK1/3 inhibitor tofacitinib could successfully reduce itch in patients with chronic pruritus of unknown origin.

Baricitinib is the first oral JAK inhibitor to be recently approved by the European Medicines Agency (EMA) for the treatment of patients with moderate to severe AD. This agent selectively blocks JAK-1 and JAK-2. In two phase 3 monotherapy studies (70) and one phase 3 combination study with topical TCS (71), baricitinib significantly reduced pruritus in test patients as compared to controls (who received placebo or TCS alone) throughout the whole observation period of 16 weeks. Baricitinib monotherapy (4 mg) reduced pruritus by 36.6 and 46.9% (vs. 12 and 16.6% in controls) in BREEZE-AD1 and BREEZE-AD2, respectively, by week 16 (70). In BREEZE-AD7, in which the additional use of TCS was allowed, baricitinib (4 mg) reduced pruritus by ≥4-point NRS in 52 and 44% of the patients (vs. 11 and 20% in placebo) at 4 weeks and 16 weeks, respectively (71). The rapid onset of itch reduction after baricitinib provision was recognized as a remarkable feature of this agent, with this onset occurring as early as 2 days after initiating treatment (71). The primary outcome parameters in these studies (i.e., the reduction of IGA and EASI) were also significantly met. Baricitinib (4 mg) not only reduced itch, but also significantly reduced sleep disturbance and improved quality of life, both of which are important patient-oriented outcome measures that improve the overall quality of life in AD patients. As a final bonus, baricitinib also significantly reduced skin pain (70, 71).

Other JAK inhibitors are currently in the pipeline for AD treatment. The most advanced in their developmental programs are upadacitinib and abrocitinib, both of which are considered selective JAK-1 inhibitors. In a recent phase 2b trial, upadacitinib reduced pruritus significantly and rapidly in moderate to severe AD patients, with a maximal itch reduction of 70% at the highest dose (i.e., 30 mg) as compared to a 10% reduction in the placebo group. In this study, eczema was also significantly reduced (72). The data from phase 3 trials will be published soon. Similarly, in a recent phase 3 trial, abrocitinib significantly reduced itch in moderate to severe AD patients aged ≥12 years. At the highest dose, abrocitinib (200 mg) effected a ≥4-point reduction in pruritus in 57% of patients (vs. 15% in the placebo group) within 12 weeks; it also significantly improved atopic eczematous skin lesion. Abrocitinib (200 mg) had already significantly reduced pruritus by the first day after starting treatment (73, 74). It will be interesting to see the not-yet-published results of a recent trial that directly compares abrocitinib with dupilumab.

One outstanding phenomenon observed in all these studies with JAK-1 or JAK-1/2 inhibitors is the speed of onset in itch reduction, which the patients experienced already within the first days of treatment. This rapid improvement in pruritus is probably due to the inhibition of several pruritic mediators (e.g., IL-4, IL-13, IL-31, and TSLP) by inhibiting their intracellular signal transduction. Together with a rapid improvement in sleep quality and overall quality of life, the patients' motivation to continue the oral treatment with JAK inhibitors increases. This is an important consideration, as *per-se* daily oral treatments are subject to lower treatment compliance as compared with subcutaneous injections of biologics every 2–4 weeks.

### Other Systemic Treatments

#### Alarmins

Although, the alarmins (e.g., TSLP and IL-33) are thought to influence AD pathophysiology, a clinical phase 2 trial in which TSLP was blocked with the specific antibody tezepelumab did not result in convincing improvements in eczema or pruritus (75). In addition, phase 2 trials with monoclonal antibodies against IL-33 were prematurely terminated due to their insufficient effects on AD. Simply because significant effects have not been observed when blocking these alarmins, however, may not necessarily mean that they do not play a role in AD itch. The study design (i.e., combination treatments with TCS, patients' characteristics and stages of the disease (acute vs. chronic AD)) can significantly influence the study outcomes. Since TSLP and IL-33 are mediators in the early phases of AD, blocking these mediators could be more important in early stages or flare-ups of the disease rather than in the chronic AD patients who are included in most AD studies. A recent finding by Wang et al. also supports a differential view of itch flare-ups as compared to chronic itch in AD patients. While previous AD trials using anti-IgE therapy had yielded mixed results in AD, the authors showed that allergen exposure is capable of inducing acute itch flare-ups *via* the stimulation of basophils that carry allergen-specific IgE, eventually releasing leukotriene (LT) C4, which then activates specific CysLTR2 receptors on sensory nerves and induces itch (76).

#### Opioid Receptor Antagonists/Agonists

With the new treatments in AD and their promising antipruritic effects, do we need more? In fact, the significant itch reduction rates of 40–70% or ≥4-point NRS reduction in about 50% of patients will already satisfy many patients. Still, many AD patients may still suffer from pruritus, and additional antipruritic “add-on treatments” for these patients may be desirable.

This goal could be reached by targeting the central and peripheral opioid system involved in chronic pruritus of AD and in end-stage renal disease (ESRD) (48). Pruritus in AD patients has been successfully reduced with the MOR antagonists naloxone or naltrexone, but the use of these agents is associated with undesirable adverse events like dizziness, drowsiness, or vomiting, hindering their broader use (49).

Newly developed KOR agonists, such as nalfurafine, or the combined MOR antagonist/KOR agonist nalbuphine have recently shown mild but significant efficacy in reducing pruritus in ESRD patients. These appear to be associated with a lower risk for central nervous adverse events (77). Currently, nalfurafine is only licensed for uremic and cholestatic pruritus in Japan. An oral extended-release formulation of nalbuphine is currently under investigation for its antipruritic effect in PN (ClinicalTrials.gov Identifier: NCT03497975). Difelikefalin (previously CR845), a peripheral KOR agonist that is intravenously applied in a dose of 0.5 μg per kg thrice weekly for 12 weeks, demonstrated significant antipruritic effects in ESRD patients under hemodialysis without displaying any dysphoria or hallucinations as adverse events (78). Oral difelikefalin, in doses of 0.25, 0.5, or 1.0 mg twice weekly, is currently under investigation for its effect on pruritus in moderate to severe AD (ClincialTrial.gov Identifier NCT04018027).

Once these drugs have been approved for the treatment of chronic pruritus in AD or chronic prurigo, it will be very interesting to see if these drugs can be used to further reduce pruritus in patients who are been given biologics or JAK inhibitors, but who are not yet free of pruritus.

### Topical Antipruritic/Anti-Inflammatory Substances

Systemic therapies for moderate to severe AD appear to have developed appreciably in recent years. However, most AD patients do not have a severe form of the disease, and many with mild to moderate forms of the disease have only circumscribed eczema but still suffer from severe pruritus. In addition, many patients do not want to be systemically treated, regardless of the severity of their disease, for various reasons. Thus, in the future topical agents will still play roles in the anti-inflammatory and antipruritic treatment of AD.

Until recently, TCS and TCI were the only “specific” topical treatments for AD. Crisaborole, a phosphodiesterase four (PDE4) inhibitor, was licensed for topical treatment of AD in 2016. Inhibition of PDE4 increases cAMP in targeted cells and reduces inflammatory mediators, eventually reducing eczema and itch associated with AD lesions. In phase 3 clinical trials, crisaborole already reduced itch significantly within the first 8 days of treatment, and the reduction remained significant throughout the 4-week study period. In addition, a significant reduction in atopic skin lesions was observed (79, 80). Although, the difference between crisaborole and placebo in reducing itch was not overwhelming, crisaborole was only associated with minor adverse events (e.g., burning or stinging), and did not cause skin atrophy.

Other new agents to treat AD are in clinical trials or already licensed. Topical JAK inhibitors are especially promising candidates as anti-inflammatory and anti-pruritic topical treatments in inflammatory skin diseases, such as AD and psoriasis. Recent studies with topical formulations of tofacitinib (a JAK1/3 inhibitor), ruxolitinib (a JAK1/2 inhibitor), and delgocitinib (a pan-JAK inhibitor that blocks all members of the JAK family) have shown promising results in reducing both eczema and especially pruritus in AD lesions (81). Delgocitinib has recently been approved for the topical treatment of AD in Japan (82). Topical JAK inhibitors are advantageous for AD patients with circumscribed pruritic AD lesions, because they can be used to control itch and the disease effectively in patients with mild to moderate AD, but also avoid the possible adverse events associated with the use of systemic JAK inhibitors (81).

Another interesting newly developed agent is tapinarof, a selective agonist for the aryl hydrocarbon receptor (AhR), also known as the dioxin receptor. Stimulation of AhR results in a decreased expression of pro-inflammatory cytokines, enhancement of the skin barrier function and a reduction in oxidative stress (83). Medicinal coal tar and soybean tar Glyteer, which have been used as anti-inflammatory agents to treat AD and psoriasis, also activate this receptor (84). In a recent phase 2b study, tapinarof 1% cream significantly reduced itch in adolescent and adult AD patients and reduced eczema (85). Significantly more patients experienced ≥3 points of NRS reduction at weeks 4 and 12 after starting the treatment, and a clear differentiation between the tapinarof and vehicle groups was shown, starting at week 2. In adult psoriasis patients, tapinarof cream also improved psoriasis lesions and significantly reduced itch (86).

## Conclusion

Chronic pruritus is the most burdensome symptom experienced by patients with AD of all grades of severity. Pruritus is the primary cause of significant impairments in the quality of life of affected patients, impacting their well-being in multiple ways. The chronic itching associated with the disease can disturb the patients' sleep and reduce their performance in their private and professional lives. It can even have significant, negative psychological consequences, such as increased anxiety and depression. The high out-of-pocket and healthcare costs associated with the treatment of pruritus and eczema puts an additional economic burden on AD patients and communities. The advent of new and effective treatments for AD promises significant improvements in care options for AD patients in the near future. Every new topical or systemic agent that has proven anti-eczematous and anti-pruritic effects will help us improve our understanding of AD pathophysiology. The improved understanding and further investigations into the anti-eczematous and anti-pruritic effects of AD treatments will also enable us to customize our therapy to meet the needs of our AD patients in the present and the future.

## Author Contributions

The author confirms being the sole contributor of this work and has approved it for publication.

## Conflict of Interest

FJ Legat reports receiving: Travel support fees from AbbVie, Bayer, Celgene, Galderma, Jansen-Cilag, Leo Pharma, Eli Lilly, Novartis, Pelpharma, and Pfizer; Lecture fees from AbbVie, Almirall, Bayer Healthcare, Celgene, Eli Lilly; Advisory board or consultant fees from Almirall, Celgene, Eli Lilly, Menlo Therapeutics, Novartis, Pfizer, Trevi Therapeutics, Vifors Pharma.
